# Inhaled Formoterol-Fluticasone Single Inhaler Therapy in Asthma: Real-World Efficacy, Budget Impact, and Potential to Improve Adherence

**DOI:** 10.1155/2020/8631316

**Published:** 2020-09-14

**Authors:** V. Rajesh, Jolsana Augustine, R. Divya, Melcy Cleetus

**Affiliations:** Department of Pulmonary Medicine, Rajagiri Hospital, Chunangamvely, Aluva, Kochi, Kerala, India

## Abstract

Asthma is the commonest chronic disease affecting airways in humans and has an increasing global disease burden. Inhaled corticosteroids (ICS) are the first-line therapeutic option for asthma, and addition of a long-acting beta 2-agonist (LABA) has been shown to improve asthma control. A combination of the two agents in a single inhaler is beneficial with regard to ease of administration and patient compliance. Various ICS-LABA formulations are available across various countries in the world, one among them being formoterol-fluticasone. Both formoterol and fluticasone have pharmacologic peculiarities which places the combination in a uniquely advantageous position when it comes to asthma therapy. The present review focuses on some of the, hitherto, less explored aspects of this combination inhaler such as real-world efficacy, impact on budget allocation, results of switch-over therapy, and potential to improve adherence to asthma treatment. It also provides practical recommendations on positioning it in real-world asthma management.

## 1. Introduction 

The Global Initiative for asthma defines asthma as a heterogeneous disease characterised by airway inflammation [1]. Asthma's prevalence is increasing in many countries, and asthma has evolved into a major health problem that affects all the age groups, a phenomenon that is universally noticed. Inhaled corticosteroids (ICS) have become the mainstay of asthma treatment [2] with a short-acting beta 2-agonist (SABA) often prescribed for immediate symptom relief. However, many patients treated with ICS and SABA have inadequate asthma control [1]. In such patients, addition of a long-acting b2-agonist (LABA) has been shown to improve asthma control [3], and hence, the ICS-LABA combination has become the norm of therapy in asthma treatment guidelines [1, 4]. The development and prescription of a fixed-dose combination (FDC) of ICS-LABA improves patient compliance. The single inhaler combination also reduces the risk of symptom-guided isolated bronchodilator overuse and ICS therapy discontinuation. The FDC of ICS-LABA offers the additional charm of reducing the health care costs associated with separate drug inhalers and has been shown to reduce respiratory-related deaths and life-threatening episodes [5, 6]. Many different FDCs of ICS/LABA combination therapies are now available across the globe. An FDC of fluticasone propionate and formoterol fumarate (FF) has been developed and has become commercially available for asthma treatment. FF single inhaler therapy is a promising option in asthma patients. It has been suggested that the bronchodilator effects of formoterol reduce the need for rescue medication and result in an increased number of episode-free days when compared with patients receiving an alternate LABA, Salmeterol [7, 8]. The FF combination has also been compared with the formoterol-budesonide inhaler [9]. Differences in the efficacy and safety among ICS/LABA combinations and choices of therapy have been previously reviewed [10, 11]. The present review focuses on some of the, hitherto, less explored aspects of this combination inhaler such as real-world efficacy, impact on budget allocation, results of switch-over therapy, and potential to improve adherence to asthma treatment. It also provides practical recommendations on positioning it in real-world asthma management.

## 2. Formoterol-Fluticasone Single Inhaler: Available Evidence and Knowledge Gaps

FF delivered via a single inhaler brings together unique peculiarities that may prove valuable in the treatment of asthmatic subjects. Both fluticasone propionate (FP) and formoterol have pharmacologic properties which places the combination in a uniquely advantageous position when it comes to asthma therapy. FP has high topical anti-inflammatory activity [12, 13] and a rapidly induced protective effect [14]. Its systemic availability is only from absorption via the lungs, whereas for the other ICS such as beclomethasone, budesonide, and flunisolide, oral bioavailability must also be taken into account [15]. FP has more potency than budesonide, beclomethasone, and flunisolide with regard to corticosteroid action in the respiratory tree [16]. Formoterol (LABA) has a rapid onset of action [17]. Salbutamol, terbutaline, salmeterol, and formoterol are the common beta 2 agonist medications used in the treatment of asthma in an inhaled form. Differences in their pharmacological properties account for differences in their clinical actions [18]. Formoterol is a full agonist at the beta 2 receptor site and has a very quick onset of action as compared to salmeterol which is a partial agonist and causes bronchodilation with higher latency. Both drugs are long-acting, but formoterol has higher intrinsic activity than salmeterol. The attribute of rapid bronchodilation is due to reasonable water solubility and adequate lipophilicity of Formoterol which ensures faster diffusion into the smooth muscle.

Many clinical studies have demonstrated the superiority of FF to either component administered as a monotherapy or concurrently via separate inhalers [19, 20]. Similarly, the FF combination has been proven to have similar efficacy and safety profiles compared to budesonide/formoterol and fluticasone-salmeterol [9, 21]. FF offers an additional benefit of rapid bronchodilation than fluticasone-salmeterol [7, 8].

Some of the less-focused aspects of FF combination therapy include the following:

1. The real-world efficacy of the FF inhaler
2. The effect of switch over of therapy from other ICS-LABA combinations (such as salmeterol-fluticasone) to FF
3. The potential of FF to improve adherence to asthma therapy
4. The efficacy of delivering FF via specialized delivery devices as opposed to a metered dose inhaler
5. The impact of using an FF inhaler on health care budget consumption

Randomized placebo-controlled trials are regarded as the highest level of evidence in evaluating drug efficacy by the scientific world. However, it is well recognised that RCTs are rarely representative of the patient populations likely to receive the same medication as treatment, and the quality of care seldom reflects what would be received in the real world. Hence, real-world observational studies in the postauthorization phase of drug development have also been undertaken. These attempts provide a means to study and better understand medicine safety, prescribing practices and adherence to guidelines in real-life clinical practice. These aspects are being addressed in the present review. The review also provides practical recommendations on positioning the formoterol-fluticasone single inhaler in real-world asthma management.

## 3. Real-World Efficacy and Safety of FF

As previously mentioned, RCTs are seldom representative of the patient populations likely to receive the same medication as treatment, and the quality of care seldom reflects what would be received in the real world. Backer et al. [22] conducted a postauthorization safety study in eight European countries. FF was prescribed as per local guidelines. This was an observational study spanning 12 months in outpatients with asthma aged ≥12 years. 2539 patients with a mean age of 47.7 years were followed up. AEs were observed in 60.0% of the patients, although the researchers concluded that only 10.2% had AEs possibly related to the FF combination. The common adverse events included asthma exacerbation (2.0% patients), dysphonia (1.8%), and cough (1.1%)]. No serious AEs were considered possibly related to FF. Based on the Asthma Control Test (ACT) score ≥20, the proportion of patients with controlled asthma increased from 29.4% at baseline to 67.4% at the study end (last observation carried forward). The percentage of patients experiencing at least one severe exacerbation decreased to 9.8% during the study as compared to 35.8% which was in the year prior to enrolment. There were also improvements from baseline in the Asthma Quality of Life scores.

The longest and the largest real-world data on the safety of FF inhaler comes from the UK primary care database [23]. Safety data for 3 years after initiation of therapy are available. The primary safety outcome was a composite of all adverse outcomes (i.e., the total accumulative number of adverse events occurring in the patient's record) for each analysis group that occurred after initiation on an FDC ICS/LABA. More than 45000 asthma patients receiving ICS-LABA were followed up of which 5727 received FF. Most of the AEs noted were mild, and the rate of AEs observed with FF were lower than that with comparator ICS-LABA formulations.

Similar observations have been made by Mansur et al. [24] in an open-label study assessing the safety and efficacy of FF spanning 12 months. This study was conducted in moderate to severe asthmatics (FEV1 40–85%) aged >12 years. The study concluded that FF is an efficacious and safe option for treating asthma. The most common AEs (>2%) of which majority were mild to moderate included nasopharyngitis, dyspnea, pharyngitis, and headache. The study drug-related AEs reported by patients were only 18 (3.8%).

## 4. Switch-Over and Step-Down Therapy with FF

Most asthma guidelines suggest step up of asthma therapy with inadequate asthma control and step down in cases of sustained good control. Switch over of therapy to a new ICS-LABA combination has been infrequently studied. However, a few studies have explored this relatively grey arena in asthma also. The effect of switch over to FF from salmeterol-fluticasone along with step down of FF dose in the next phase has been better examined by Usmani et al. [25]. This 24-week pragmatic open-label RCT was attempted in well-controlled asthmatics already on a salmeterol-fluticasone inhaler. The phase 1 of the study consisted of a 12-week change phase to FF from salmeterol-fluticasone; proceeding to the second phase was carried out only in those patients who retained asthma control following the first phase. This involved a step down of the FF dose. 225 subjects in phase 1 were randomized in a 2 : 1 fashion to FF and fluticasone-salmeterol; of these, 116 remained stable on FF and were further randomized in a 1 : 1 fashion to same dose vs. dose step down. The primary end point was the 7-question Asthma Control Questionnaire (ACQ7) score. Patients tolerated the switch of therapy and step down of FF dose particularly well without worsening asthma control.

The real-world effectiveness and cost impact of switch over to FF from fluticasone-salmeterolhas also been studied [26]. A historical matched cohort database study from the UK assessed for the noninferiority of the FF cohort of patients (initiating treatment with FF or changing from salmeterol-fluticasone to FF) with the fluticasone-salmeterolcohort (comprising patients initiating and remaining on salmeterol-fluticasone pMDI combination therapy). Noninferiority of effectiveness was defined as the prevention of severe exacerbations. After a 1 : 3 matching, a total of 2472 patients were studied (618 in the FF cohort and 1854 in the fluticasone-salmeterol cohort. The proportion of severe exacerbation-free patients in the FF cohort was not inferior to salmeterol-fluticasone cohort, and therapy was accomplished at a lower average annual cost compared with the salmeterol-fluticasone cohort. A real-world study from India on FF administered via a specialized device has been addressed in a subsequent section.

## 5. Potential of the FF Single Inhaler to Improve Treatment Adherence and Asthma Control

Table 1 provides a short summary of the trials on FF with regard to real-world efficacy, budget impact, and effect of switch over. There is lack of direct evidence as to whether utilization of FF will improve treatment adherence in asthma. However, the combination has attributes that might theoretically improve therapy adherence and asthma control. The rationale behind combining fluticasone with formoterol was to provide the benefits of a high-potency topical anti-inflammatory agent along with rapid onset of bronchodilator action in a new formulation single inhaler. A single inhaler device combining the two agents has multiple benefits. It ensures adequate symptomatic relief hand-in-hand with anti-inflammation. The need to use two drugs or two inhalation devices separately is circumvented. Finally, the risk of bronchodilator overuse without ICS use is avoided. Patients appreciate drugs which act quick and have sustained action; hence, while prescribing ICS/LABA combination therapy, the potency of the ICS and the speed of onset of the LABA are considered crucial factors by the clinicians [31–35]. Therefore, patients and physicians may give priority to an inhaled therapy containing components with the abovementioned attributes. It may be mentioned that the potential for FF to improve inhaler adherence may be evaluated in a randomized trial structured to this end.

## 6. Inadequate Asthma Control: Focus on India

Although widely perceived as an easy-to-control disease, chronic asthma control remains suboptimal despite the availability of efficacious molecules for the treatment of asthma. This is true across the globe with no geographic area being spared [36–39]. Asthma control in the real-world remains an elusive goal for clinicians and patients in India. A survey conducted in North America, Europe, the Asia-Pacific region, and Latin America by the Asia Pacific Asthma Insight and Management (AP-AIM) reported that none of the asthmatic patients in India (*n* = 400) had guideline-defined asthma control [25]. Additionally, Indian patients had the highest exacerbation frequency, the highest numbers of overnight hospitalizations, and the largest proportion of respondents who had to miss work or school because of asthma.

**Figure 1:**
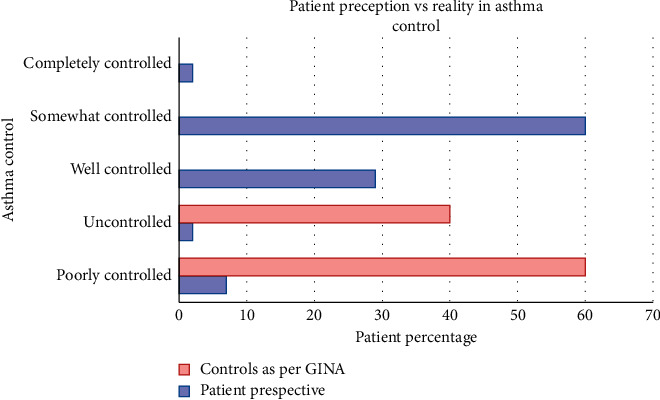
Control of asthma: patient perception versus GINA guideline-defined control.

**Figure 2:**
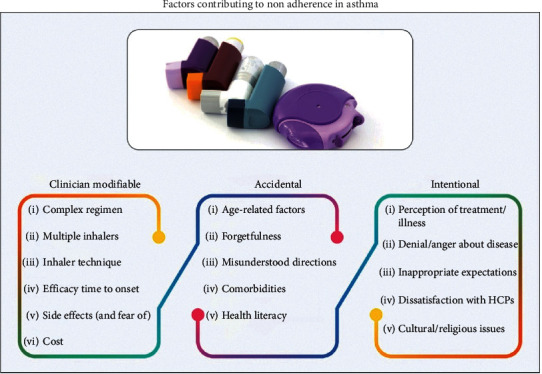
Factors contributing to medication nonadherence in asthma.

The causes of inadequate asthma control as evidenced in published Indian studies have been manifold. There has been an inadequacy on the part of clinicians themselves in adequately prescribing controller medications [40]. Proper demonstration of the inhaler technique and communication skills are vital for successful asthma control with inhaler therapy, which has previously been reported to be lacking [40, 41]. Sociocultural beliefs of patients do play a role in medication adherence, and many patients believe that inhaled medications might prove addictive [42]. Patients tend to overestimate their level of asthma control and can be poor symptom perceivers.  Figure 1 depicts the patient-perceived asthma control versus asthma control in reality as observed in studies. Ease and effectiveness of the inhaler delivery device has crucial implications in achieving compliance to therapy [43]. Controller medications with slow onset of action may be perceived as ineffective by patients, and noncompliance rates may be higher [44]. The availability of different combinations and formulations of ICS and LABA may provide patients with unique and individually tailored treatment options according to the clinical severity and device preferences. This flexibility translates to increasing probability of compliance to the treatment and effectiveness of therapy [45, 46]. The common causes of noncompliance to inhaled medications have been summarized in Figure 2.

## 7. Administration of FF via Specialized Devices

As previously stressed, the choice of inhaler has a major bearing on patient satisfaction, compliance to treatment, asthma control, and exacerbation rate. The efficacy and safety of FF delivered via the Revolizer device have been assessed in a multicentre Indian study by Ghoshal et al. [29]. This was a prospective, open-label, noncomparative, real-world observational study carried out in 15 centers across India. The patients were followed up for 24 weeks. The study population comprised of adult patients (aged above 18 years) with persistent asthma who were either already taking FF combination capsules (100/6 mcg or 250/6 mcg) through the Revolizer or were uncontrolled on other treatments and required a change in their treatment to FF FDC as per the treating physician's discretion. The mean change in the ACT outcome at 4, 8, 16, and 24 weeks was considered as the primary efficacy outcome. Secondary efficacy analyses included peak PEFR (morning and evening), number of patients having symptom-free days and nights, and the number and severity of exacerbations episodes in the 24 weeks' time period. Response to the Usability Preference Satisfaction Confidence questionnaire after 1 week was also noted down. Study subjects showed an improvement in the ACT score and all other outcome measures. 94% of the patient population expressed good satisfaction with the Revolizer device.

Delivery of FF via the K-haler (a novel breath-triggered inhaler - BTI) was studied by Bell et al. [30] in a randomized, open-label, two-period, crossover study. Adolescent and adult patients with both asthma and COPD were recruited and examined regarding their ability to correctly handle the FFpMDI or FFK-haler. The patient had to follow a simple, standardized training regimen. The primary endpoint was the ability to perform all the steps correctly at the first use. An identical proportion (77.2% versus 72.1%) of 307 patients performed all the steps correctly while using pMDI and K-haler BTI, whereas the corresponding proportions performing all critical steps correctly were 82.4% and 87.0%, respectively. An anumerically greater proportion found the pMDI easier to use than either the Turbuhaler or Accuhaler DPIs. The preference data and ease of use challenged the perceived notion that dry powder inhalers (DPI) are necessarily simpler to use. The study has two drawbacks. First, outcome measures such as asthma control, lung functions, or exacerbations were not measured. Second, the inclusion of both asthma and COPD patients makes the mix too heterogeneous to draw stern conclusions.

Notwithstanding the efficacy of these specialized delivery devices, emerging data suggest that the flow rate dependence for drug deposition that is well described forCFC pMDIs [47–49] may be less applicable to HFA. In vitro data have revealed that FF HFA has a consistent fine particle fraction of around 40% at flow rates of 30–60 L/min [50], whereas functional respiratory imaging data have demonstrated consistent total lung deposition of 36%–44% with either a sharp or gradual inspiratory profile and an inspiratory flow rate of 30 or 60 L/min [51]. These data imply that FF HFA may ensure reasonable and constant drug deposition with variation in inspiratory flow rate than the earlier generation of CFC pMDIs and specialized delivery devices may not be necessarily superior.

## 8. Budget Impact of the FF Single Inhaler

Budget allocation to health care delivery and medications is of vital concern to health departments, and most, if not all, state governments will prefer cheaper therapy as long as clinical results are not compromised. Analyses have been conducted in the UK and Spain on the budget impact of using a FF single inhaler in asthma as opposed to using other ICS-LABA combination. Dunlop et al. [28] estimated the annual budget impact for the UK NHS when using FP-FF as an alternative to fluticasone-salmeterol.Cost involved in drug acquisition, administration, and monitoring was determined for the combinations. The scenario analyses reviewed varying rates of uptake, adherence, AE-related costs, and resource use associated with switching treatment. Results revealed that annual drug purchase costs per person were lower with FP-FF (£412) than with fluticasone-salmeterol (£509). This translated to a potential annual savings of a huge amount (£15,110,279) to the NHS. Similar results were replicated 3 year later in another study from the UK [27]. Total annual costs per person year was less with FF (£625) as compared to fluticasone-salmeterol (£734). For all scenarios with increased FP-FF prescription volumes, the annual total costs to the NHS decreased.

Analysis was conducted in Spain also with a similar objective [52]. FF was economical to acquire than fluticasone-salmeterol or budesonide-formoterol (20% and 30%, respectively). The cost per patient in the FF cohort was 9326€/year, which was 1.5% and 2.6% cheaper than fluticasone-salmeterol and budesonide-formoterol, respectively. These impacts on budgeting may not be applicable in India, where formoterol-budesonide is marketed at a much lower price than fluticasone-salmeteroland FF inhalers. However, we do not have formal analyses of this sort conducted in the Indian subcontinent to date.

## 9. Summary and Conclusions

FF brings together a time-honored LABA (having a rapid onset and longer duration of action) and a potent ICS (good topical potency and low systemic bioavailability) in a single-aerosol inhaler. Previous trials have demonstrated the superiority of FF to either component administered as a monotherapy or concurrently via separate inhalers. Similar efficacy and safety profiles of FF as compared to budesonide/formoterol and fluticasone-salmeterol are also well established, and an additional benefit offered by FF is of rapid bronchodilation than fluticasone-salmeterol. Further to these established facts, studies critically appraised in this review suggest that the FF inhaler has excellent efficacy in real world, good tolerance of switch over of therapy from other ICS-LABA, positive impact on budget allocation/health care costs, and good delivery via HFA MDI and specialized devices. Although the impact of these advantages on adherence to therapy has not been formally evaluated, these attributes may encourage patients to better comply to their treatment regimen, a factor that has been always associated with real-world improvements in asthma control [53, 54]. To summarize, the single inhaler FF combination aerosol critically appraised in this review represents an additional therapeutic option for the treatment of asthma in adolescents and adults who require an ICS/LABA, with properties that may place it as the number one option in clinical practice in these patient subsets.

## Figures and Tables

**Table 1:** Summary of clinical studies on the FF single inhaler.

Study/year	Country	No. of subjects (*n*)	Design	Outcome
Studies comparing switch in therapy of FF with salmeterol-fluticasone				
Usmani et al. [25]	England	225	A randomized, controlled, pragmatic, open-label trial on well-controlled asthmatic patients	In patients with well-controlled asthma, a change from fluticasone-salmeterol to FF did not compromise asthma control. Step down of FF was well tolerated
Simon et al. [26]	The UK	2472	A historical, matched cohort database study evaluated two treatment groups in the Optimum Patient Care Research Database in the UK	Changing to, or initiating FF combination therapy, is associated with a noninferior proportion of patients who are severe exacerbation-free at a lower average annual cost compared with continuing or initiating treatment with fluticasone-salmeterol

Long-term real-world studies of efficacy and safety				
Backer et al. [22]	Czech republic, Denmark, France, Ireland, Norway, Slovak Republic, Sweden, and the United Kingdom	2539	A 12-month observational study of outpatients with asthma	In this real-world postauthorization safety study, FF demonstrated a safety profile consistent with that seen in controlled clinical trials
Price et al. [23]/2019	The UK	41,609	A historical, longitudinal cohort database study using UK primary care data from the Clinical Practice Research Datalink (CPRD) database	FF was associated with an overall lower adverse outcome rate
Mansur et al. [24]	Germany, Hungary, Poland, Romania, and the United Kingdom	413	An open-label study, mild to moderate-severe asthmatics	FF had a good safety and efficacy profile over the 6- and 12-month study periods

Budget impact analysis of FF				
Emily et al. [27]	The United Kingdom	—	Real-world analysis	The use of FF as an alternative to fluticasone-salmeterol can result in cost savings for the NHS when assessing drug
Dunlop et al. [28]	The United Kingdom	—	Real-world analysis	The comparable efficacy and lower acquisition costs of FF compared with fluticasone-salmeterol make it a cost-saving option for the UK NHS for the treatment of asthma patients requiring combination maintenance therapy using a pMDI

Studies on FF using specialized inhalation devices				
Ghoshal et al. [29]	India	385	A prospective, open-label, noncomparative, real-world observational, 24-week, multicenter study	FF FDC capsules administered via a single-dose DPI, (Revolizer®) offer a novel, well-tolerated, and effective treatment option for the long-term management of asthma
Bell et al. [30]	The United Kingdom	307	A randomized, open-label, two-period, crossover study	Ease of use and preference data for FF pMDI challenged the perceived wisdom that DPI are necessarily simpler to use, whereas the corresponding data for FF K-haler strongly favoured this novel BTI over the Turbuhaler, Accuhaler, and other pMDIs
